# Medical Department of Niagara University

**Published:** 1889-04

**Authors:** 


### *MEDICAL DEPARTMENT OF NIAGARA UNIVERSITY.*

### Close of the Winter Session—Spring Course of Medical Lectures.

The medical faculty of Niagara University have organized a course of didactic, clinical and laboratory instruction, under a corps of able instructors, to open April 15th, and to continue six or seven weeks. This movement practically makes the medical course eight and a half months in length, and enables the faculty to include many studies which it has been found impossible to reach in the regular course of six months. We desire to witness a further advance in this excellent school of medicine, by extending the term to nine months. This college has been in the vanguard of medical reform in this State, and these movements are healthy indications of the spirit actuating its growing body.

The Winter course closes with the commencement exercises, which will be held April 9th, at which a small class of thoroughly-equipped young men will graduate. It may be stated that the examinations extending over a period of two weeks, will be severe, though fair and impartial, and the institution will fully sustain its reputation for thoroughness.

In organizing the supplementary or Spring course, the faculty have borne in mind the fact that students of medicine, in prosecuting their studies, need wiser direction and guidance than the preceptor can furnish. Besides, the laboratory facilities provided are ample, enabling the students to devote time to departments which demand greater exactions than he can afford in the regular session.

We are confident this movement will be supported by the profession, who will send their students just commencing the study of medicine, and thus furnish them with facilities necessary in the four months of their pupilage.

